# Enhanced visible light photocatalytic activity of Gd-doped BiFeO_3_ nanoparticles and mechanism insight

**DOI:** 10.1038/srep26467

**Published:** 2016-05-20

**Authors:** Ning Zhang, Da Chen, Feng Niu, Sen Wang, Laishun Qin, Yuexiang Huang

**Affiliations:** 1 College of Materials Science and Engineering, China Jiliang University, Hangzhou 310018, Zhejiang, P.R. China

## Abstract

To investigate the effect of Gd doping on photocatalytic activity of BiFeO_3_ (BFO), Gd-doped BFO nanoparticles containing different Gd doping contents (Bi_(1−x)_Gd_x_FeO_3_, x = 0.00, 0.01, 0.03, 0.05) were synthesized using a facile sol-gel route. The obtained products were characterized by X-ray diffraction, scanning electron microscopy, transmission electron microscopy, X-ray photoelectron spectra, and ultraviolet-visible diffuse reflectance spectroscopy, and their photocatalytic activities were evaluated by photocatalytic decomposition of Rhodamine B in aqueous solution under visible light irradiation. It was found that the Gd doping content could significantly affect the photocatalytic activity of as-prepared Gd-doped BFO, and the photocatalytic activity increased with increasing the Gd doping content up to the optimal value and then decreased with further enhancing Gd doping content. To elucidate the enhanced photocatalytic mechanism of Gd-doped BFO, the trapping experiments, photoluminescence, photocurrent and electrochemical impedance measurements were performed. On the basis of these experimental results, the enhanced photocatalytic activities of Gd-doped BFO could be ascribed to the increased optical absorption, the efficient separation and migration of photogenerated charge carriers as well as the decreased recombination probability of electron-hole pairs derived from the Gd doping effect. Meanwhile, the possible photocatalytic mechanism of Gd-doped BFO was critically discussed.

Photocatalytic oxidation has been widely proven as one of the advanced oxidation processes for pollutant degradation^1^. Many oxide semiconductors, such as TiO_2_, ZnO, and SnO_2_, have been proven as efficient photocatalysts for photocatalytic degradation of organic pollutant and photocatalytic water splitting owing to their excellent ultraviolet absorbency, relatively high photocatalytic activity, robust chemical stability, low cost and nontoxicity^2^. However, these oxide semiconductors having a wide band-gap can only absorb about 5% of sunlight in the ultraviolet region, which greatly limits its practical applications. Thus, the development of visible-light-driven photocatalysts has recently become a very important topic of research.

Over the past few years, perovskite bismuth ferrite (BiFeO_3_, denoted as BFO) has attracted great interest owing to its outstanding high Curie temperature (T_c_ ≈ 1100 K) and G-type antiferromagnetic order below the Neel temperature (T_N_ ≈ 643 K) properties^3^. These characteristics make it become one of good candidates for room-temperature multiferroic materials, which are attractive for applications in magnetic recording media, light-emitting diodes and spintronic devices^4^,^5^,^6^. Recently, BFO has also been demonstrated as a promising visible-light driven photocatalyst because of its suitable band gap (~2.2 eV), good chemical stability and intrinsic electric polarization field^7^,^8^,^9^. Nevertheless, the reported photocatalytic activity of BFO is still low because of the rapid recombination of photogenerated electron–hole pairs in BFO^10^. Therefore, strategies are needed for improvement of the photocatalytic activity of BFO.

It is accepted that the recombination of photogenerated electron-hole pairs could be inhibited by creating electron- or hole-trapping centers within the lattice of the photocatalyst^11^. In this context, doping of rare earth in the photocatalyst has been considered as an effective approach to improve the photocatalytic activity^12^, because the rare earth dopants within the lattice of the photocatalyst could be acted as the electron or hole trapping sites thus to facilitate the production and separation of photogenerated electron-hole pairs during the photocatalytic reaction^13^. To date, doping of BFO with a foreign atom (such as Dy, Sr, Mn) at either A or B site of the ABO_3_ lattice has proven effective for enhancing its photocatalytic properties^14^,^15^,^16^. For example, Sakar *et al*.^14^ demonstrated that substitution of Bi^3+^ with Dy^3+^ resulted in remarkable improvement of the photocatalytic activity of BFO under visible light irradiation. Though there have been several reports on doping of transition metal or rare earth in BFO, the research towards the doping of rare earth in BFO for photocatalytic applications is still at an early stage, and a number of challenges remain to be explored. For example, the intrinsic mechanism for the enhanced photocatalytic activity of rare earth-doped BFO still remains unclear and needs to be explored further.

Recently, it has been reported that the photocatalytic activity of BFO was enhanced by the doping of Gd, and the enhanced photocatalytic activity was considered as a consequence of the increased ferroelectric domains in Gd-doped BFO^17^,^18^. From the perspective of photoelectrochemistry, however, the mechanism insight into the influence of Gd doping on the photocatalytic activities of BFO has not yet been clarified. Therefore, this work aims to elucidate the enhanced photocatalytic mechanism of Gd-doped BFO in depth from the point view of photoelectrochemistry. In this work, the Gd-doped BFO nanoparticles were synthesized by a facile sol-gel method, and the photocatalytic activities of the as-prepared samples were then evaluated by photocatalytic degradation of rhodamine B (RhB) under visible light irradiation. Various photoelectrochemical techniques, such as the trapping experiments, photoluminescence, photocurrent and electrochemical impendence measurements, were for the first time employed to explore the enhanced photocatalytic mechanism of Gd-doped BFO composites. On the basis of these experimental results, a possible photocatalytic mechanism of Gd-doped BFO was critically discussed.

## Results and Discussion

### Materials characterization

**Figure 1:**
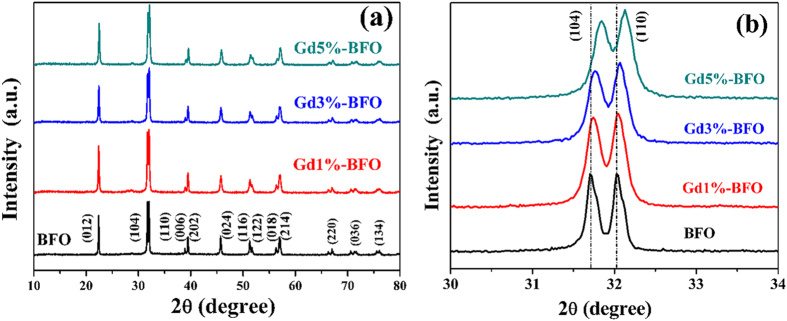
(**a**) XRD patterns of Bi_1−x_Gd_x_FeO_3_ (x = 0.00, 0.01, 0.03, 0.05); (**b**) the magnified patterns of Bi_(1−x)_Gd_x_FeO_3_ in the range of 30° ~ 34°.

Figure 1(a) shows the XRD patterns of as-prepared pure BFO and Gd doped BFO samples. The diffraction pattern of pure BFO was well matched with the rhombohedral structure with R3c space group (JCPDS No. 86–1518), indicating that single crystalline BFO phase could be obtained by the present sol-gel process. For the Gd-doped BFO samples, the diffraction patterns were similar to that of pure BFO, and no additional peaks for secondary impurities or other phases were observed, demonstrating a good dispersibility of Gd dopant in the BFO host. From the magnified XRD patterns in the vicinity of 2*θ* around 32 degrees (Fig. 1b), however, it can be seen that as increasing the Gd dopant content the separated (104) and (110) diffraction peaks shifted towards higher 2*θ* values obviously, arising from the substitution of smaller sized Gd^3+^ (9.38 Å) ions for Bi^3+^ (10.3 Å) ions in BFO^19^. This suggests that the rhombohedral structure of BFO was distorted by Gd substitution, which has also been observed in other rare earth doped BFO materials^20^. The structural distortion in BFO by Gd substitution could give rise to the contraction in lattice parameters, unit cell and volume, as confirmed by the calculated unit cell parameters in Table 1. In addition, the crystalline sizes calculated from the XRD patterns using Scherer’s formula were found to be 40.23 nm, 32.18 nm, 27.81 nm and 27.71 nm for pure BFO, Gd1%-BFO, Gd3%-BFO and Gd5%-BFO, respectively. With increasing the Gd doping content, the crystalline size of Gd-doped BFO gradually decreased, further revealing the structural distortion of BFO induced by the Gd doping.

**Figure 2:**
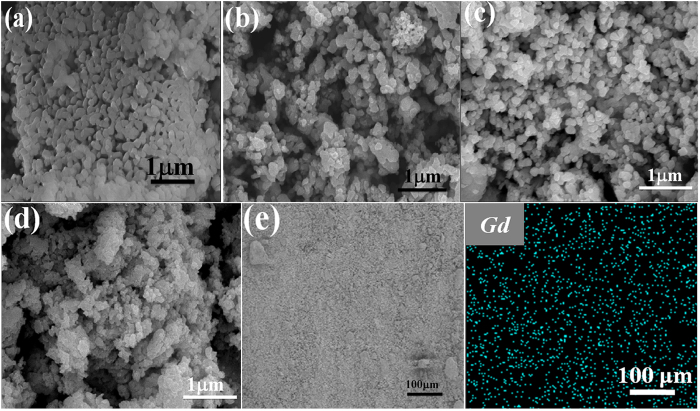
SEM images of as-prepared samples: (**a**) BFO; (**b**) Gd1%-BFO; (**c**) Gd3%-BFO; (**d**) Gd5%-BFO; (**e**) Gd elemental mapping of Gd3%-BFO sample; and (**f**) EDS spectra of Gd3%-BFO sample. (*Note*: the labeled elements of carbon (C) and platinum (Pt) in the EDS pattern should be detected from the conductive tape and sprayed metal during the specimen preparation for SEM measurement, respectively).

**Figure 3:**
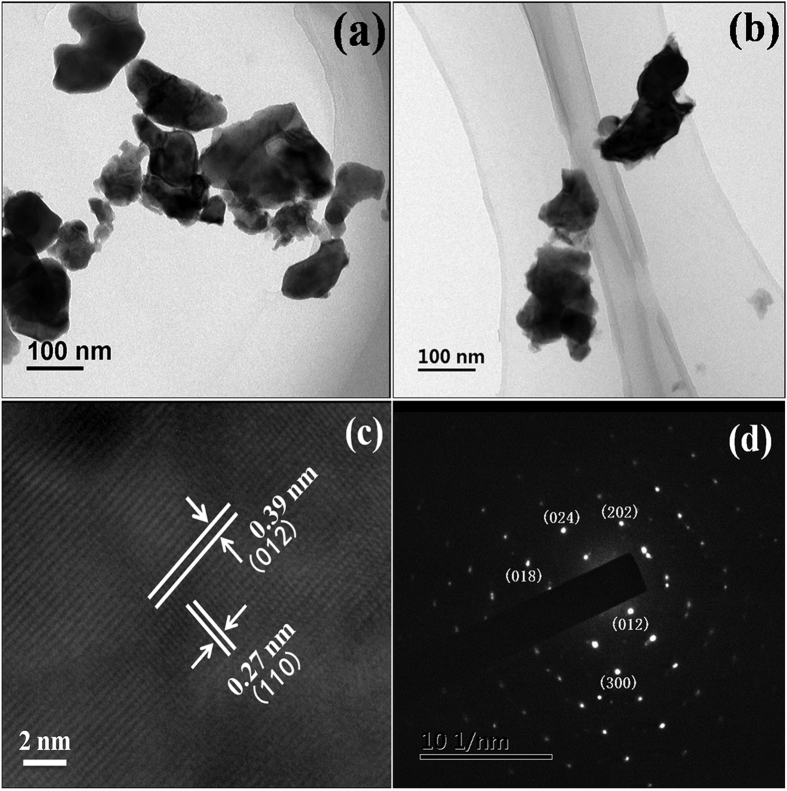
TEM images of the (**a**) pure BFO nanoparticles and (**b**) Gd3%-BFO sample; (**c**) HRTEM micrograph and (**d**) SAED pattern of Gd3%-BFO sample.

The SEM images of as-prepared pure BFO and Gd-doped samples are given in Fig. 2(a–d). As shown, the pure BFO particles were irregular and prone to aggregate with an average particle size of ca. 150 nm. For Gd-doped BFO samples, the particles were also irregular. Upon the increment of Gd dopant content from 1% to 5%, the particle size was found to be gradually reduced, which was in agreement with the calculated lattice parameters given in Table 1. The observed size reduction with increasing Gd dopant content could be ascribed to the substitution of the smaller sized of Gd^3+^ ions for Bi^3+^ ions in BFO. The smaller particle size in most cases would give rise to a larger surface area and stronger adsorption capacity, which is usually beneficial for photocatalytic activity^21^. In addition, the EDS spectrum of Gd3%-BFO (Supplementary Figure S1) indicates that the sample was composed of Bi, Fe, O, and Gd elements, and the atomic ratio of Gd to (Bi + Gd) was about 3.8% in approximately consistent with the feeding ratio. Meanwhile, the Gd elemental mapping (Fig. 2(e)) reveals the uniform distribution of Gd dopant in the BFO host. To further determine the actual Gd doping contents in the as-prepared photocatalysts, the inductively coupled plasma mass spectrometry (ICP-MS) measurements were performed. The ICP-MS results are summarized in Table 2. As seen, the actual Gd doping contents in the as-prepared photocatalysts were detected to be 0, 0.99%, 2.87% and 4.76% for BFO, Gd1%-BFO, Gd3%-BFO and Gd5%-BFO, respectively, which were very close to the theoretical Gd doping contents, confirming the successful Gd doping in the Gd-doped BFO samples. Figure 3 shows the TEM and high resolution TEM (HRTEM) images of pure BFO and Gd3%-BFO samples. As shown, both the pure BFO (Fig. 3(a)) and the Gd3%-BFO samples (Fig. 3(b)) were irregular particles, and the particle size of Gd3%-BFO was smaller than that of pure BFO, which was in consistent with the SEM results. The HRTEM image for the Gd3%-BFO sample (Fig. 3(c)) identifies the crystal lattice fringes with two different interplanar distances of 0.27 nm and 0.39 nm, which could be assigned to the (110) and (012) crystal planes of Gd3%-BFO, respectively. In addition, the representative selected-area electron diffraction (SAED) pattern (Fig. 3(d) further confirms the crystalline phase of BFO in the Gd3%-BFO sample, and the diffraction spots ((018), (024), (202), (012) and (300)) manifest a high order of crystallinity and systemic lattice orientation in the Gd3%-BFO particles.

**Figure 4:**
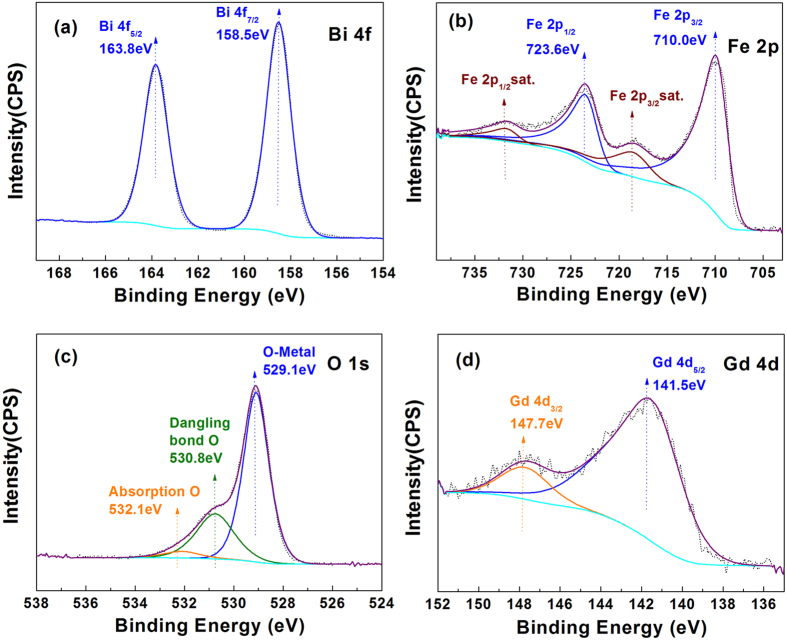
XPS spectra of (**a**) Bi, (**b**) Fe, (**c**) O, and (**d**) Gd elements for the Gd3%-BFO sample.

To determine the chemical states of Bi, Fe, O, and Gd elements in the Gd3%-doped BFO nanoparticles, the XPS technique was employed. The obtained spectrum for Bi 4f is shown in Fig. 4(a). The two characteristic peaks for Bi 4f appeared at around 158.5 eV and 163.8 eV corresponding to Bi 4f_7/2_ and Bi 4f_5/2_, respectively, indicating that bismuth existed in the form of Bi^3+^ in the Gd-doped BFO^22^. As shown in Fig. 4(b), the binding energies of Fe at 710.0 eV and 723.6 eV corresponding to the Fe 2p_3/2_ and Fe 2p_1/2_ were attributed to a spin orbital interaction. The occurrence of a satellite peak at 718.2 eV (≈8.0 eV above the Fe 2p_3/2_ peak) indicates that the Fe element was in the Fe^3+^ valence state in the Gd3%-BFO sample^23^,^24^. The O 1s XPS spectrum (Fig. 4(c)) shows that the three peaks positioned at 529.1 eV, 530.8 eV and 532.1 eV could be ascribed to oxygen–metal bonds, dangling bonds and surface-adsorbed oxygen, respectively^25^. The XPS spectrum for Gd 4d (Fig. 4(d)) reveals that the characteristic Gd 4d peaks centered at 141.5 and 147.7 eV could be attributed to Gd 4d_5/2_ and Gd 4d_3/2_, respectively, confirming the presence of Gd^3+^ valence state in the Gd-doped BFO samples^19^. The XPS spectra further confirm the successful preparation of Gd-doped BFO nanoparticles.

**Figure 5:**
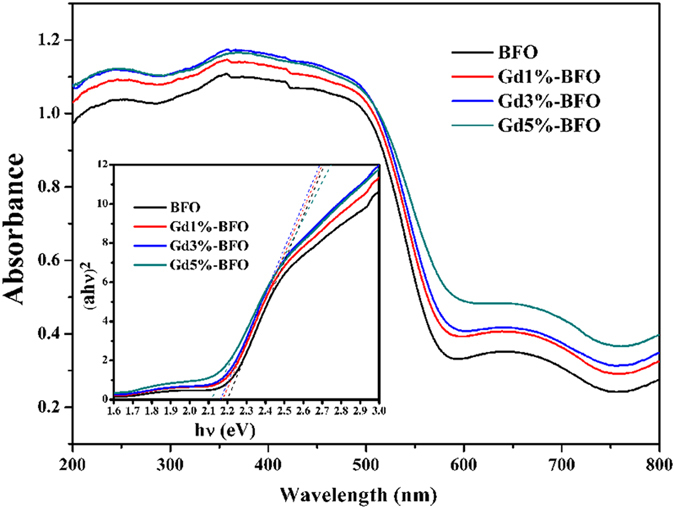
UV-vis diffuse reflectance spectra of as-prepared pure BFO and Gd-doped BFO samples. Insets are the plots to determine the band gaps for the each sample.

To investigate the influence of Gd doping on the optical absorption of BFO, the UV-vis DRS spectra of pure BFO and Gd-doped BFO samples were measured at room temperature, as shown in Fig. 5. The absorption band edge of BFO nanoparticles appeared at 563 nm, which was similar to those previously reported^26^, indicating that BFO could respond to visible light for photocatalytic reaction. Compared to pure BFO, the Gd-doped BFO samples exhibited enhanced absorption capability especially in the visible light region, and the absorption intensity became gradually stronger as increasing the Gd dopant content. Moreover, the band gap could be calculated from the plot of the Kubelka-Munk function^7^,^27^ ((αhν)^2^ vs photon energy (hν)) for the direct band gap semiconductor, as presented in the inset of Fig. 5. The band gaps were estimated to be 2.2, 2.18, 2.16, 2.1 eV for the pure BFO, Gd1%-BFO, Gd3%-BFO and Gd5%-BFO samples, respectively. With the increase of the Gd dopant content, the band gap of doped samples was gradually decreased, in turn leading to a higher absorption capability. Undoubtedly, the enhanced absorption property of Gd-doped BFO would probably improve the photocatalytic activity of BFO, as discussed below.

### Photocatalyic performances

**Figure 6:**
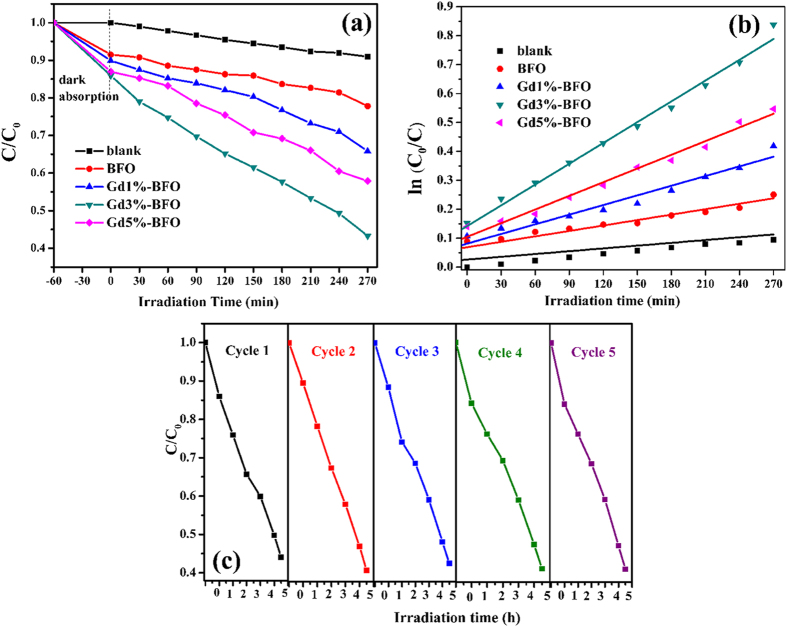
(**a**) Photocatalytic degradation of RhB as a function of the irradiation time under visible light for the as-prepared pure BFO and Gd-doped BFO samples; (**b**) Pseudo-first order kinetics fitting data for the photodegradation of RhB; (**c**) Photocatalytic degradation of RhB with the Gd3%-BFO sample for five cycles.

The photocatalytic activities of the as-prepared samples were evaluated by photocatalytic degradation of model pollutant RhB. Figure 6(a) shows the photocatalytic degradation efficiency of RhB catalyzed by pure BFO and Gd-doped BFO photocatalysts. When reaching the equilibrium adsorption state in the dark, pure BFO, Gd1%-BFO, Gd3%-BFO and Gd5%-BFO could adsorb approximately 8.6%, 10.2%, 14.1%, and 13.1% of RhB molecules, respectively. The enhanced equilibrium RhB adsorption of Gd-doped BFO samples might be the result of the reduced particle size of Gd-doped BFO as well as the presence of much more surface defects in Gd-doped BFO samples induced by the Gd doping. Under visible light irradiation, the pure BFO photocatalyst could decompose 22.3% of RhB after 270 min irradiation time. When Gd-doped BFO samples were used as the photocatalyst, a significant increase of RhB photodegradation efficiency was observed. After 270 min visible light irradiation time, 34.2%, 56.8% and 42.1% of RhB were decomposed for the Gd1%-BFO, Gd3%-BFO and Gd5%-BFO samples, respectively. The Gd3%-BFO sample yielded the highest RhB degradation efficiency, which was about 2.55 times that of the pure BFO. To quantitatively analyze the reaction kinetics of RhB degradation, the experimental data in Fig. 6(a) could be fitted by the Langmuir–Hinshelwood model, as expressed by the following equation (Equation 1)^28^:


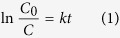


where *C*_0_ and *C* are the concentrations of RhB at different irradiation time of *t*_0_ and *t*, respectively, and *k* is the pseudo-first-order rate constant of photodegradation (min^−1^). From the linear fitting curves of ln(*C*_0_/*C*) versus irradiation time *t* (Fig. 6(b)), the RhB degradation rate constant *k* were calculated to be 3.6 × 10^−4^, 5.45 × 10^−4^, 1.07 × 10^−3^, 2.38 × 10^−3^, and 1.55 × 10^−3^ min^−1^ for the blank sample, pure BFO, Gd1%-BFO, Gd3%-BFO and Gd5%-BFO, respectively. Among them, the Gd3%-BFO sample exhibited the highest *k* value, which was 4.37 times that of pure BFO. The photocatalytic experiments reveal that the doping of Gd could significantly enhance the photocatalytic activity of BFO photocatalyst.

As known, the stability and reusability of a photocatalyst is an important parameter for practical applications. To evaluate the stability and reusability of Gd-doped BFO photocatalyst, the Gd3%-BFO photocatalyst was recycled 5 runs for photodegradation of RhB, as depicted in Fig. 6(c). After 5 successive runs with each reaction lasting for 270 min, the degradation efficiency of Gd3%-BFO photocatalyst could be largely maintained, indicating a good stability. This result also confirms that the RhB degradation by Gd-doped BFO photocatalyst was photocatalytic reaction rather than photo-corrosive one.

### Photocatalytic mechanism

The photocatalytic process usually involves three steps: (1) the absorption of photons with energy larger than the band gap of a photocatalyst, (2) the generation, separation, migration or recombination of photogenerated electron–hole pairs, and (3) the redox reactions on the photocatalyst surface. As discussed above, the Gd-doped BFO samples exhibited much higher photocatalytic activities than pure BFO sample. The enhanced photocatalytic activities of Gd-doped BFO could be closely related to the following three aspects: (1) the extension of excitation wavelength, (2) the decrease of charge carrier recombination, and (3) the promotion of surface redox reactions. The extension of excitation wavelength for Gd-doped BFO was verified by the above-mentioned DRS measurements. Compared to the pure BFO sample, the absorption coefficients for Gd-doped BFO samples were elevated to some extent in both visible and ultraviolet light region. Based on the Kubelka-Munk function, the gradual decrease in the band gap was expected for Gd-doped BFO samples with the increase of Gd doping content, indicating effective narrowing of band gap and much stronger light absorption features. In this regard, Gd doping in BFO lattice would greatly enhance the light harnessing capability of BFO, which could be beneficial for photocatalytic activity.

**Figure 7:**
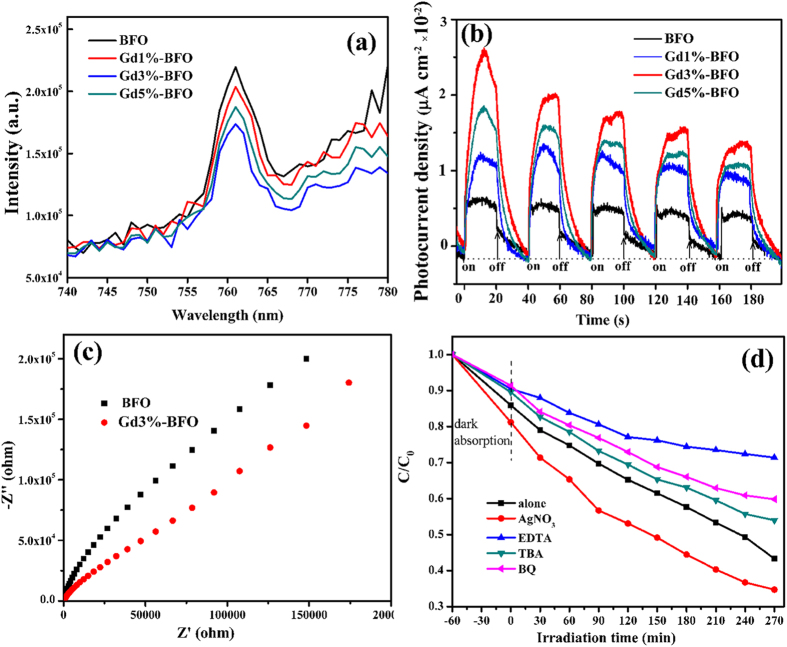
(**a**) PL emission spectra of the as-prepared pure BFO and Gd-doped BFO samples at an excitation wavelength of 400 nm; (**b**) Photocurrent responses of as-prepared BFO, Gd1%-BFO, Gd3%-BFO, and Gd5%-BFO samples electrodes with light on/off cycles under visible light irradiation (λ ≥ 420 nm) at 0 V vs SCE in 0.5 M Na_2_SO_4_ aqueous solution; (**c**) EIS spectra for the as-prepared pure BFO and Gd3%-BFO samples in 0.5 M Na_2_SO_4_ aqueous solution under visible light illumination (λ ≥ 420 nm); (**d**) Photocatalytic degradation of RhB over the Gd3%-BFO sample alone, and with the addition of different types of active species scavengers (i.e., AgNO_3_, EDTA, TBA and BQ).

Another important factor that influences the photocatalytic activity is the photogenerated carrier behaviors in the photocatalyst. It is believed that the photocatalytic activity is largely dependent on the generation, separation, transport and recombination of photogenerated electron–hole pairs in the photocatalyst^29^. To elucidate the enhanced photocatalytic mechanism of Gd-doped BFO samples, the separation, migration, and recombination process of photogenerated electron–hole pairs in the photocatalysts were investigated by various photoelectrochemical techniques including PL, photocurrent and EIS measurements. Figure 7(a) shows the PL spectra of pure BFO and Gd-doped BFO photocatalysts. It can be observed that the emission band for pure BFO was centered at 762 nm, and the position of emission peaks for Gd-doped BFO photocatalysts was similar to that of pure BFO. The PL intensity decreased successively in the order of BFO, Gd1%–BFO, Gd5%–BFO, and Gd3%–BFO, indicating that the Gd-doped BFO samples could capture photoinduced electrons more efficiently than pure BFO. It is known that the PL emission intensity is related to the recombination of excited electrons and holes, and the lower emission intensity is indicative of a decrease in recombination probability^30^. Among all the prepared photocatalyst samples, the Gd3%-BFO sample had the lowest PL intensity, indicating the lowest recombination probability of excited electrons and holes in the photocatalyst which would contribute significantly to the highest photocatalytic activity (Fig. 6). It should be noted that the Gd5%-BFO sample showed a higher PL emission intensity than the Gd3%-BFO sample. This could be ascribed to the fact that the excess amount of Gd dopants in the Gd5%-BFO sample would probably produce much more surface defects, which could capture the photo-induced electrons to further produce excitons^31^, thus leading to the enhanced PL emission intensity. In addition, the transient photocurrent response of a photocatalyst may directly correlate with the separation and transport efficiency of the photogenerated carriers^32^. Figure 7(b) shows the transient photocurrent spectra for pure BFO and Gd-doped BFO samples, which were measured via several on/off cycles under visible light irradiation (λ ≥ 420 nm). Both pure BFO and Gd-doped BFO samples responded quickly to the on/off visible light irradiation, and the transient photocurrent was obtained accordingly. Compared to pure BFO, Gd-doped BFO samples exhibited much higher photocurrents, indicating a much more efficient photoinduced charge separation and transfer process and a longer lifetime of the photogenerated charge carriers in the Gd-doped BFO sample. Among all the samples, the Gd3%-BFO photocatalyst produced the highest photocurrent intensity, implying that the Gd3%-BFO photocatalyst possessed the maximal photo-quantum efficiency and thus would achieve the highest photocatalytic activity. Furthermore, the interface charge separation efficiency was also investigated using the EIS measurement. Figure 7(c) shows the typical EIS spectra for pure BFO and Gd3%-BFO photocatalyst under visible light irradiation (λ ≥ 420 nm). The arc radius in the impedance spectrum can reflect the reaction rate on the photocatalyst surface, and the smaller arc radius implies a more effective separation of photogenerated electron–hole pairs and a higher efficiency of charge transfer across the electrode/electrolyte interface^33^. As shown, the arc radius of the Gd3%-BFO sample electrode was smaller than that of pure BFO electrode, suggesting that the Gd-doped BFO structure could facilitate the separation and migration of photogenerated electron-hole pairs in comparison with pure BFO, which was in agreement with the photocurrent result.

From the above-mentioned PL spectra, photocurrent and EIS measurements, it can be seen that there was an optimum dosage of rare earth ions (Gd^3+^) in BFO particles for the most efficient separation and migration of photogenerated electrons and holes, which could be explained in terms of the space charge layer thickness^34^. As known, the value of the space charge layer thickness for the effective separation of photogenerated charge carriers must not be lower than a value^35^ and the thickness of space charge layer can be affected by dopant content according to the following equation (Equation 2)^36^:


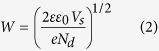


where *W* is the thickness of the space charge layer, *ε* and *ε*_0_ are the static dielectric constants of the semiconductor and of the vacuum, *V*_s_ is the surface potential, *N*_d_ is the number of dopant donor atoms, *e* is the electronic charge. With the increase of the dopant content (*N*_d_), the thickness of space charge layer (*W*) would decrease^37^. Consequently, there should be an optimum rare earth dopant content (*N*_d_), which would make the thickness of space charge layer substantially equal to the light penetration depth^38^. In our case, as the Gd dopant content increased towards the optimal value, the surface barrier became higher thus making the space charge region narrower, and hence the electron–hole pairs within the region would be separated efficiently. When the Gd dopant content was over its optimum, the space charge region would become much narrow, and the penetration depth of light into the photocatalyst would exceed the space charge layer. Under this circumstance, the recombination of the electron-hole pairs would become easier, thus making the photocatalytic activities of the photocatalyst decrease. Therefore, there was an optimum Gd dopant content in Gd-doped BFO samples for the best photocatalytic activity.

In addition, one more factor that significantly influences the photocatalytic activity involves the redox reactions on the photocatalyst surface, where organic pollutants would be eventually decomposed through photocatalytic oxidation by some reactive species such as h^+^, ·OH and 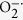
39. To elucidate the photocatalytic mechanism, the trapping experiments were performed to determine the main reactive species of Gd-doped BFO for the RhB degradation. The photocatalytic degradation of RhB by the Gd3%-BFO photocatalyst was carried out with the addition of different quenchers under visible light irradiation, and the results of the trapping experiments are given in Fig. 7(d). It can be seen that the photodegradation efficiency of RhB with the addition of tert-butyl alcohol (TBA, a hydroxyl radicals (∙OH) scavenger^40^, 2 mM) or benzoquinone (BQ, a superoxide radicals (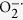) scavenger^41^, 0.5 mM) was slightly decreased compared to those without addition of quenchers, and the addition of ethylene diamine tetraacetic acid (EDTA, a hole scavenger^42^, 2 mM) resulted in remarkable suppression of RhB photodegradation activity. When AgNO_3_ (an electron scavenger^43^, 2 mM) was added, however, the degradation efficiency significantly increased due to the consumption of photo-generated electrons by AgNO_3_. This means that the consumption of photo-generated electrons could increase the separation efficiency of photo-generated electron–hole pairs. The trapping experiments reveal that the predominant active species for RhB photodegradation by the Gd-doped BFO catalyst were photogenerated holes (h^+^), superoxide radicals (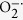), or hydroxyl radicals (∙OH) rather than photogenerated electrons.

On the base of the above experimental results, a possible mechanism for the enhanced photocatalytic degradation of RhB by the Gd-doped BFO photocatalyst was proposed as follows. The Gd-doped BFO particles could be excited by visible light (λ ≥ 420 nm) to produce photogenerated electrons and holes (Equation 3). The rare earth (Gd) dopant in Gd-doped BFO would serve as electron traps^11^,^13^ which could capture excited electrons (Equation 4) and facilitate the separation of electron–hole pairs, thus promoting the charge transfer from the bulk BFO to the surface of the photocatalyst. Consequently, the photoinduced electrons transferred from the Gd dopant to the photocatalyst surface could capture the adsorbed O_2_ and reduce it to 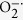 (Equation 5), and the formed 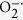 would further participate the RhB degradation reactions (Equation 8). Simultaneously, the photogenerated holes after transporting to the photocatalyst surface could also react with H_2_O to form ·OH (Equation 6) for the degradation of RhB (Equation 9) or directly oxidize RhB (Equation 7). According to the trapping experiments, holes and superoxide radicals were believed to be the predominant reactive species for RhB degradation (Equations 7 and 8), while hydroxyl radicals could also play minor roles in the degradation process (Equation 9). Eventually, the organic pollutant could be mineralized into CO_2_, H_2_O, or inorganic ions. The proposed photocatalytic mechanism of Gd-doped BFO for RhB degradation could be described as follows:


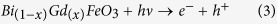



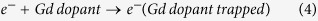



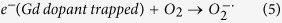



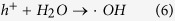



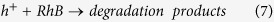



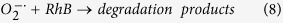



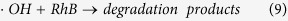


## Conclusion

In this work, a series of Gd-doped BFO photocatalysts containing different Gd dopant contents were prepared by a facile sol-gel method. The successful substitution of Gd^3+^ in BFO was confirmed by various characterization techniques such as XRD, SEM, TEM, and XPS. Compared to pure BFO, the Gd-doped BFO samples exhibited enhanced absorption capability especially in the visible light region. The band gap of Gd-doped BFO samples was gradually decreased with the increase of the Gd dopant content. The photocatalytic activities of the prepared photocatalysts were evaluated by photocatalytic degradation of RhB under visible light irradiation. It was found that Gd-doped BFO photocatalysts exhibited much higher photocatalytic activity than pure BFO, and the highest photocatalytic activity was obtained for the Gd3%-BFO sample. The enhanced photocatalytic activities of Gd-doped BFO could be ascribed to the increased optical absorption, the efficient separation and migration of photogenerated charge carriers as well as the decreased recombination probability of electron-hole pairs derived from the Gd doping effect. In addition, the photocatalytic mechanism for Gd-doped BFO photocatalyst was also proposed.

## Methods

### Preparation of Gd doped BFO nanoparticles

All chemicals were of analytical grade without further purification. Gd-doped BFO nanoparticles with a substitution formula of Bi_1−x_Gd_x_FeO_3_, where x = 0, 0.01, 0.03, or 0.05, were synthesized by a simple sol-gel method, and were named as BFO, Gd1%-BFO, Gd3%-BFO and Gd5%-BFO, respectively. Typically, the precursor solution was prepared by mixing appropriate amounts of bismuth nitrate hydrate (10 × (1 − x) mmol), iron nitrate hydrate (10 mmol) and gadolinium nitrate hexahydrate (10 × (x) mmol) in the desired stoichiometric ratio dissolved in 100 mL of ethylene glycol. Tartaric acid, with 1 : 1 stoichiometric ratio with respect to the metal nitrates, was added to the above solution to obtain a homogeneous sol. The sol was then heated up to ~80 °C to obtain a dried gel, which was subsequently grinded into powders. Finally, the as-obtained powders were annealed at 550 °C for 2 h to obtain Gd-doped BFO nanoparticles.

### Materials characterizations

The crystalline phases of as-prepared samples were characterized by X-ray powder diffraction (XRD) on Bruker D2 X-ray diffractomer using Cu *K*_α_ radiation (λ = 1.5418 Å). The morphology and chemical composition of the samples were characterized by scanning electron microscopy (SEM, Hitachi SU 8010) attached with energy dispersive X-ray Spectroscope (EDS), and element mapping was also taken for the chemical analysis of the sample. The microstructures were also observed by a transmission electron microscopy (TEM) on the JEM-2100 electron microscopy (JEOL, Japan) with an accelerating voltage 200 kV. The actual Gd doping contents of as-prepared photocatalysts were determined using inductively coupled plasma mass spectrometry (ICP-MS, PE NexION 300X). The chemical states of the prepared samples were investigated by using X-ray photoelectron spectroscopy (XPS). The spectra were recorded on a PHI 5000 Versa Probe with Al *K*_α_ radiation. Ultraviolet-visible (UV-vis) diffuse reflectance spectra (DRS) of the samples were recorded on a Shimadzu UV-3600 in the wavelength range of 200 ~ 800 nm equipped with an integrating sphere, and BaSO_4_ was used as a reflectance standard. The photoluminescence (PL) measurements were recorded on a Hitachi High-Tech F-7000 fluorescence spectrophotometer with a xenon lamp at the excitation wavelength of 400 nm.

### Photocatalytic measurements

The photocatalytic activities of as-prepared BFO and Gd-doped BFO samples were evaluated by photodegradation of RhB in aqueous solutions under visible light irradiation (*λ* ≥ 420 nm) using a 300 W Xe lamp accompanied with a cutoff filter as a light source. The initial concentration of RhB was 5 mg/L. In each experiment, 0.3 g of the photocatalyst was suspended in an aqueous solution (100 mL) of RhB in a quartz glass reactor, which was cooled by refluxing water to get rid of any thermal catalytic effect. Prior to irradiation, the suspension was stirred in the dark for 1 h to achieve the adsorption-desorption equilibrium. At given time intervals, 4 mL of suspension was collected and centrifuged at 9000 rpm for 30 min to remove the catalyst powders. The concentration of RhB was then detected by measuring the maximum absorbance at 554 nm using a spectrophotometer (model 722, Precision Instrument Co., Ltd. Shanghai, China). As for the stability test, the remaining photocatalyst powders after photocatalytic degradation of RhB in suspension were collected by centrifugation, washed with distilled water to remove the residual RhB, and then dried before another photocatalytic reaction. This process was recycled five times.

### Photoelectrochemical measurements

For the photoelectrochemical measurements, the working electrode with an active area of 1 × 1 cm^2^ was prepared by doctor blading of a slurry containing 90% obtained sample and 10% polymer binder (polyvinylidene difluoride) onto the surface of ITO conductive glass and then dried in a vacuum oven at 60 °C for 12 h. Photocurrent measurements were performed on an electrochemical workstation (CHI660D, CH Instruments, China) in a three-electrode quartz cell with a 0.5 M Na_2_SO_4_ electrolyte solution, using the fabricated sample electrode as the working electrode, a platinum wire as the counter electrode, and a standard Hg/Hg_2_Cl_2_ (saturated KCl) as the reference electrode. Electrochemical impedance spectra (EIS) were also obtained in the same three-electrode configuration with a sinusoidal ac perturbation of 10 mV.

## Additional Information

**How to cite this article**: Zhang, N. *et al*. Enhanced visible light photocatalytic activity of Gd-doped BiFeO_3_ nanoparticles and mechanism insight. *Sci. Rep*. **6**, 26467; doi: 10.1038/srep26467 (2016).

## Supplementary Material

Supplementary Information

## Figures and Tables

**Table 1:** Unit cell parameters of Bi_1−x_Gd_x_FeO_3_ compositions.

Compositions	a = b (Å)	c (Å)	Cell volume(Å^3^)
BiFeO_3_	5.5821	13.87945	374.54
Bi_0.99_Fe_0.01_O_3_	5.5816	13.86932	374.21
Bi_0.97_Fe_0.03_O_3_	5.5792	13.85718	373.55
Bi_0.95_Fe_0.05_O_3_	5.5711	13.83003	371.74

**Table 2:** The actual and theoretical Gd doping contents in the BFO and Gd-doped BFO samples.

Catalysts	ICP-MS (Gd doping content)		Theoretical values (Gd doping content)	
	Weight ratio (mg/Kg)	Molar ratio (%)	Weight ratio (mg/Kg)	Molar ratio (%)
BFO	0	0	0	0
Gd1%-BFO	5026	0.99	5036	1
Gd3%-BFO	14520	2.87	15159	3
Gd5%-BFO	24115	4.76	25349	5
