# Supplementary material for: Enhanced visible light photocatalytic activity of Gd-doped BiFeO3 nanoparticles and mechanism insight
Source: Sci Rep. 2016 May 20;6:26467. doi: 10.1038/srep26467 (PMC4873739; doi:10.1038/srep26467)
Supplement: Supplementary Information [file srep26467-s1.doc]

**Supporting information**

**Enhanced visible light photocatalytic activity of Gd-doped BiFeO_3_ nanoparticles and mechanism insight**

Ning Zhang, Da Chen*, Feng Niu, Sen Wang, Laishun Qin*, Yuexiang Huang

*College of Materials Science and Engineering, China Jiliang University, Hangzhou 310018, Zhejiang, P.R. China*

*Tel: +86-571-86835738, Fax: +86-571-86835740*

*Email:* *dchen_80@hotmail.com; qinlaishun@cjlu.edu.cn*


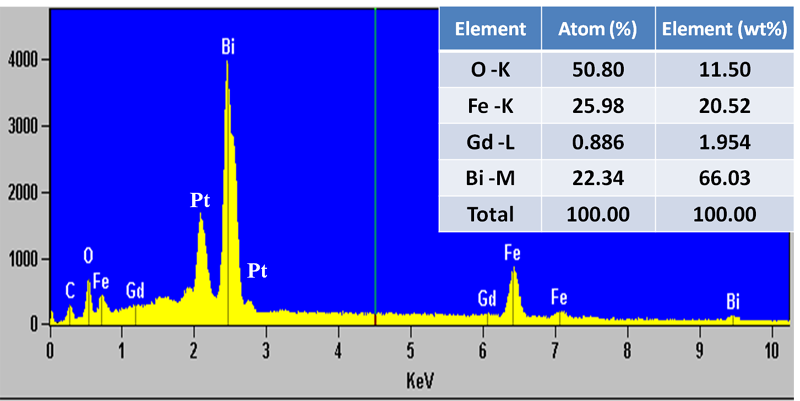


**Figure S1.** EDS spectra of Gd3%-BFO sample. (*Note*: the labeled elements of carbon (C) and platinum (Pt) in the EDS pattern should be detected from the conductive tape and sprayed metal during the specimen preparation for SEM measurement, respectively.)
